# North American and European practices for opioid-sparing and opioid-free anaesthesia: a cross-sectional survey

**DOI:** 10.1016/j.bjao.2025.100511

**Published:** 2025-12-15

**Authors:** Yann Gricourt, Nancy M. Boulos, Yann Daoulas, Tristan Grogan, Brenton Alexander, Myriam Mezzarobba, Philippe Cuvillon, Esther M. Pogatzki-Zahn, Helene Beloeil, Maxime Cannesson, Patrice Forget, Alexandre Joosten

**Affiliations:** 1 Department of Anesthesiology and Perioperative Medicine, Nimes University Hospital, Nimes, France; 2 Department of Anesthesiology & Perioperative Medicine, David Geffen School of Medicine, University of California Los Angeles, Los Angeles, CA, USA; 3 IMAGINE UR UM 103, Montpellier University, Anaesthesia Critical Care, Emergency and Pain Medicine Division, Nîmes University Hospital, Nîmes, France; 4 Department of Anesthesiology and Intensive Care Medicine, Lapeyronie University Hospital, Montpellier, France; 5 Department of Medicine Statistics Core, David Geffen School of Medicine, University of California Los Angeles, Los Angeles, CA, USA; 6 Department of Anesthesiology & Perioperative Medicine, University of California San Diego, La Jolla, CA, USA; 7 Department of Biostatistics, Epidemiology, Public Health and Innovation in Methodology, CHU Nimes, IDESP, INSERM, University of Montpellier, Nimes, France; 8 Department of Anesthesiology, Intensive Care and Pain Medicine, University Hospital Muenster, Muenster, Germany; 9 University of Rennes, CHU Rennes, Inserm, OSS 12142, CIC 1414, Anaesthesia and Intensive Care Department, Rennes, France; 10 Aberdeen Centre for Arthritis and Musculoskeletal Health (Epidemiology Group), Institute of Applied Health Sciences, School of Medicine, Medical Sciences and Nutrition, Aberdeen, UK; 11 Anaesthesia Department, NHS Grampian, Aberdeen, UK; 12 Pain and Opioids after Surgery (PANDOS) European Society of Anaesthesiology and Intensive Care (ID ESAIC_RG_PAND) Research Group, Brussels, Belgium

**Keywords:** analgesia, dexmedetomidine, intraoperative management, ketamine, multimodal analgesia, opioid-free anaesthesia, opioid crisis, regional anaesthesia

## Abstract

**Background:**

Opioids remain central to perioperative analgesia but concerns about the growing opioid crisis and adverse effects have prompted revaluation of their role. Opioid-sparing anaesthesia and opioid-free anaesthesia (OFA) have emerged as alternatives, yet their clinical adoption remains uncertain. This survey assessed adoption and perceptions among anaesthesiologists in North America and Europe.

**Methods:**

A 26-question cross-sectional, web-based survey was distributed via email to members of the American, European, and French Societies of anaesthesiologists. The survey assessed routine use of opioid-sparing techniques, defined as the regular use of non-opioid analgesics and adjuncts to minimise intraoperative opioid use in the past month. We hypothesised that fewer than 50% of anaesthesiologists routinely used these techniques during this period.

**Results:**

The overall response rate was 2% among ASA members (614/31 000) and 12% among European Society of Anaesthesiology and Intensive Care (ESAIC) members (414/3500). Concern about opioid use was reported as high in ESAIC and ASA members (90% *vs* 83%, *P*<0.001). Daily use of opioid sparing techniques was reported by 37% (95% confidence interval [CI] 32–42%) of ESAIC and 40% (95% CI 36–45%) of ASA members. OFA use was less common overall but reported to be higher by ASA repondents (21%, 95% CI 18–25%) *vs* 12% (95% CI 9–15%), *P*<0.001) for EASIC respondents. Perceived risks differed: EASIC respondents more often cited haemodynamic instability (43% *vs* 16%, *P*<0.001), whereas ASA respondents more often cited patient dissatisfaction (55% *vs* 30%) and uncontrolled pain (72% *vs* 53%, both *P*<0.001). Key barriers to OFA adoption included limited training, low confidence, and lack of evidence-based guidelines.

**Conclusions:**

Interest in opioid-sparing anaesthesia and OFA is widespread, but routine use remains modest and varies by region. Regional perceptions, institutional protocols, and confidence in evidence appear to influence implementation.

Opioids have long been pivotal to intraoperative analgesia worldwide,^1^ valued for their ability to attenuate nociception and maintain haemodynamic stability. However, increasing awareness of their perioperative side-effects—including nausea, sedation, ileus, and delayed recovery—has prompted renewed interest in alternative approaches.^2^ Additionally, enhanced recovery after surgery (ERAS) protocols^3^^,^^4^ and the Perioperative Surgical Home (PSH) initiative^5^^,^^6^ now advocate for opioid-sparing strategies that integrate non-opioid analgesics, regional techniques, and multimodal approaches to optimise patient outcomes.

In parallel, societal attention to the opioid crisis, particularly in North America, has elevated the importance of judicious opioid use in all clinical settings, including the perioperative setting where postoperative overprescribing has been identified as a contributing factor.^7^ However, the contribution of intraoperative opioid use to long-term opioid-related harms remains uncertain. While some advocate for minimising opioids across the perioperative continuum, others question the impact of intraoperative reductions on meaningful clinical outcomes such as pain and patient satisfaction. As a result, opioid-sparing anaesthesia and opioid-free anaesthesia (OFA) have become areas of interest (and debate) within the anaesthesiology community. Opioid-sparing anaesthesia refers to the intraoperative use of adjuncts (e.g. NSAIDs, acetaminophen, lidocaine, dexmedetomidine, regional techniques) to reduce but not eliminate opioid administration. OFA, by contrast, seeks to completely avoid opioids during anaesthesia. While both approaches have been studied in randomised trials, their adoption into routine practice has been inconsistent.

To date, no survey has explored how anaesthesia providers across major high-income regions, specifically North America and Europe, perceive and implement these strategies. Specifically, there is limited insight into how clinicians interpret key terminology, the barriers they encounter, and how their attitudes vary based on experience or practice environment.

We therefore conducted an international survey of anaesthesiologists in North America and Europe to better understand the current landscape of opioid-sparing anaesthesia and OFA. We sought to quantify the prevalence of reported daily use of opioid-sparing technique, explore attitudes toward these techniques, and identify perceived barriers and training needs.

## Methods

This survey was approved by both the ethics committee of the French Society of Anesthesiologists (IRB 00010254-2024-023) on 9 March 2024, and the University of California Los Angeles (IRB-24-5788) on 4 December 2024. Participation was voluntary and anonymous, with no incentives offered. Participants provided informed consent by clicking ‘Start Survey’ on the cover letter, which included the study objectives, data protection and contact information. This study adheres to the Checklist for Reporting Results of Internet E-Surveys (CHERRIES) guidelines^8^ (Supplementary Material S1) and Checklist for Reporting of Survey Studies (CROSS)^9^ (Supplementary Material S2)

### Survey participants

The survey was a cross-sectional, international, web-based survey using a convenience sampling strategy of members from the ASA, the European Society of Anaesthesiology and Intensive Care (ESAIC), and the French Society of Anaesthesia and Critical Care (SFAR). Additionally, to capture perspectives from low- and middle-income countries (LMIC), members of South African Society of Anaesthesiologists (SASA), the Tunisian Society of Anaesthesia, Analgesia, and Intensive Care (STAAR), and the Senegalese Society of Anaesthesia and Critical Care (SOSEAR) were also invited to participate.

Eligible participants were anaesthesia providers from these societies, including residents, fellows, attending anaesthesiologists, and certified registered nurse anaesthetists (CRNAs) in Europe. Medical students, retired practitioners, and USA CRNAs could not take part in the survey because the ASA does not maintain a CRNA membership list or email database, and thus survey invitations could not be distributed to this group.

### Survey questionnaire construction

Survey development followed the methodological framework proposed by Story and Tait.^10^ Given the absence of previous surveys on this topic, a steering committee of five international experts initially developed a questionnaire based on the BRUSO (Brief, Relevant, Unambiguous, Specific, and Objective) framework to ensure item clarity and readability.

A pilot test was then conducted among eight representative participants not involved in the study design (two residents, two CNRAs, four attendings) to assess the cover letter and questionnaire usability, completion time (estimated time <10 min), and compatibility across devices.^10^ Based on feedback, minor adjustments were made to achieve a consensus among all steering committee members.

The final instrument comprised four sections: general information, opioid-sparing anaesthesia practices, OFA practices, and education and acceptance. General information section included questions on age, clinical experience and position, primary practice environment, country of practice (with state-level details for participants from the USA), and potential subspecialties. The initial questionnaire was developed in French and then translated into English using a cross-cultural adaptation approach by two native English-speaking anaesthesiologists. Potential differences between their translations were reviewed and discussed within the steering committee. The questionnaire was available only in English and French, which allowed broad distribution within the target societies but may have limited participation from countries where these are not primary languages.

The final questionnaire (Supplementary Material S3) was made available in both English and French, depending on the targeted country. It included between seven and 26 questions, with one open-ended question for additional suggestions,^10^ displayed across four screens. Branching logic was applied to hide questions based on prior responses. For example, if respondents indicated they did not use OFA in their daily practice, related questions were hidden. All questions were compulsory with a completeness check before submission. Non-response options (e.g. “Don’t know”, ‘Neutral’, or ‘Prefer not to answer’) were provided to reduce survey dropout.^10^ Unsubmitted and incomplete surveys were excluded from the final analysis.

To ensure consistent understanding, opioid-sparing anaesthesia was explicitly defined as the use of adjuncts (e.g. NSAIDs, acetaminophen, lidocaine, ketamine, dexmedetomidine, regional blocks) to reduce but not eliminate opioid administration such as remifentanil, sufentanil, fentanyl, hydromorphone, or morphine at the beginning of its section. OFA was intentionally left undefined, as its definition was a survey question, but traditionally has been defined as when a practitioner seeks to completely avoid opioids during anaesthesia.

The survey was hosted on REDCap (Research Electronic Data Capture),^11^ a secure, web-based platform that supports validated data capture, audit trails, and automated data export. Data were protected by password access. No personal information including internet protocol (IP) addresses, names, or contact details were recorded. To prevent duplicate responses, participants were instructed in the cover letter to respond only once. An ‘Already completed’ status was displayed next to the ‘Start Survey’ button to reduce multiple participations. Cookies or IP address checks were not used for entry prevention. Participants could not return to previous pages or modify their answers once submitted but could edit responses on the current page before proceeding. Additionally, partially completed surveys could not be revisited or completed later.

### Survey dissemination

To ensure a quick and broad dissemination, the survey was promoted through email lists and online newsletters, as follows: survey invitations were sent to ASA members starting on 12 January 2025 (with two reminders spaced 1 week apart), and to ESAIC members starting on 26 January 2025 (without any reminder). In France and South Africa, the survey link was included in the respective societies’ online newsletters for one month, starting on 2 September 2024 and 10 December 2024, respectively. In Senegal and Tunisia, survey invitations were distributed via local email databases over a similar 1-month period, with two reminders sent during the campaigns, starting on 17 November 2024 and 9 January 2025, respectively. The survey was disseminated over 6 months, and data collection closed on 11 March 2025.

### Endpoints

The primary endpoint was the prevalence of daily opioid-sparing anaesthesia usage, defined as reporting this strategy at least once per day (‘frequently’) for patients undergoing general anaesthesia in the past month. Secondary endpoints included current practices for opioid-sparing anaesthesia and OFA. This encompassed knowledge, medication combinations, perceptions, barriers, local protocol implementation, and the need for guidelines or additional training. The prevalence of OFA use was defined as respondents reporting its implementation at least once a day.

### Statistical analysis

Because the true prevalence of opioid-sparing anaesthesia is unknown, we hypothesised it to be ∼30% [5%] margin of error based on expert opinion, ERAS guidelines, and the increasing adoption of multimodal analgesia and regional anaesthesia techniques. A minimum sample size of 323 participants was estimated to yield a 95% confidence interval (CI) with a total width of 0.10, assuming a sample proportion of 0.30. This corresponds to a margin of error of plus or minus 5% which is commonly considered acceptable for prevalence estimates in survey-based research. This calculation was thus based on a precision-based (margin-of-error) approach and was not directly adjusted for the total membership size of the surveyed societies. Descriptive statistics were used to summarise the characteristics of the study population. Continuous variables were summarised as means with standard deviations or medians with inter-quartile ranges, depending on distribution. Categorical variables were reported as counts and percentages. Europe *vs* USA comparisons were assessed using the χ^2^ test for categorical variables or Fisher’s exact test. For attitudinal questions, responses were collapsed into binary categories (e.g. ‘concerned’ or ‘very concerned’ *vs* all other responses), and χ^2^ tests were used to compare proportions between European and USA respondents. Missing data were documented but not imputed. For the analysis of factors associated with the use of opioid-sparing anaesthesia or OFA, univariable and multivariable logistic regression was performed. Explanatory variables (predetermined based on clinical relevance and prior literature) included gender, years of experience, practice setting (academic, non-academic public hospital, or private practice), geographic region, anaesthesia subspecialty, and the presence of institutional guidelines. Adjusted odds ratios (OR) with 95% CIs and *P*-values were reported. To provide deeper insight, a qualitative content analysis with sentiment coding (ranging from very negative to very positive) was performed using Qualtrics Text iQ for the open-ended question (Q23). This AI-based tool uses natural language processing algorithms to automatically extract sentiment and thematic content. Sentiment classifications were manually reviewed by a study team member to ensure contextual accuracy. To visualise the geographic distribution of opioid-sparing anaesthesia use, we generated a USA state-level heatmap. State responses were cleaned to standardise non-canonical entries (e.g. ‘COL’ converted to ‘CO’) and filtered to include only valid USA state abbreviations. For each state, we calculated the proportion of respondents who reported OFA use and overlaid this prevalence onto a choropleth map, with colon intensity representing OFA usage and transparency scaled to the sample size per state. State abbreviations were annotated with sample sizes to provide context for interpretation. All visualisations were performed in R version 4.4.3 using the ‘ggplot2’ package. To explore practice profiles related to OFA, we performed an unsupervised k-means clustering analysis. Clustering variables included OFA frequency (Q15), self-rated knowledge (Q14), perceived benefit and risk evidence (Q16 and Q17), training needs (Q21), reported use of i.v. agents (lidocaine, ketamine, alpha-agonists; Q18), and region (Europe *vs* USA). Variables with non-response codes (e.g. 88) were set to missing and participants with incomplete responses were excluded. All selected variables were standardised before clustering. K-means clustering was performed using three centres (k=3), with 25 random initialisations. Cluster assignments were then merged back with the original dataset for interpretation. Cluster-level summary statistics were calculated for each variable, and values were scaled from 0 to 1 to enable comparison. These standardised cluster profiles were seen using a radar (spider) plot generated with the ‘fmsb’ package in R. The plot displays each cluster’s relative use of OFA-related practices and perceptions. All statistical tests were two-sided, and a significance level of *P*<0.05 was considered statistically significant. Analyses were conducted using R V4.4.3 (R Foundation for Statistical Computing, Vienna, Austria).

## Results

Among 1156 individuals who started the survey, 1028 completed it fully (89% completion rate). The overall response rate was 2% among ASA members (614/31 000) and 12% among ESAIC members (414/3500). Responses from SFAR members were included in the ESAIC tally, as many French anaesthesiologists hold dual ESAIC–SFAR membership. Responses from African societies were not included in the main analysis because of a low response rate (73 responses); these results are presented in Supplementary Material S4. Nevertheless, we analysed these 73 African responses descriptively to provide some insight into LMIC perspectives, while acknowledging that they cannot be interpreted as representative of their regions. To avoid introducing instability into the primary prevalence estimates, their data are presented separately.

### Respondent characteristics

Overall, most respondents were male (64%), attending anaesthesiologists (60%) with >10 yr of clinical experience (64%), and affiliated with academic hospitals (53%). Forty percent of respondents did not report any specific anaesthesia subspecialty (Table 1). Significant regional differences were observed among responders (*P*<0.001), with more ASA respondents compared to ESAIC respondents having ≥10 yr of clinical experience (75% *vs* 49%) and working in private institutions (40% *vs* 12%).

**Table 1:** Participants’ characteristics. CNRA, Certified Nurse Registered Anaesthetist; ESAIC, European Society of Anaesthesiology and Intensive Care; ASA, American Society of Anesthesiologists.

Variables	Overall (*N*=1028)	ESAIC (*N*=414)	ASA (*N*=614)	*P*-value
Gender, *n* (%)				<0.001
• Male	658 (64)	235 (57)	423 (69)	
• Female	340 (33)	172 (42)	168 (27)	
• Other	5 (1)	2 (1)	3 (1)	
• Decline to state	25 (2)	5 (1)	20 (3)	
Official tittle, *n* (%)				<0.001
• CNRA	23 (2)	22 (5)	1 (0)	
• Resident	88 (9)	56 (14)	32 (5)	
• Fellow	42 (4)	39 (9)	3 (1)	
• Attending anaesthesiologist	616 (60)	240 (58)	376 (61)	
• Academic anaesthesiologist	191 (19)	36 (9)	155 (25)	
• Head of anaesthesia department	68 (7)	21 (5)	47 (8)	
Clinical experience, *n* (%)				<0.001
• In training	55 (5)	32 (8)	23 (4)	
• 0–2 yr	79 (8)	45 (11)	34 (6)	
• 3–4 yr	95 (9)	57 (14)	38 (6)	
• 5–9 yr	137 (13)	78 (19)	59 (10)	
• 10+ yr	662 (64)	202 (49)	460 (75)	
Primary practice, *n* (%)				<0.001
• Academic teaching hospital	549 (53)	288 (70)	261 (43)	
• Non-academic public hospital	184 (18)	78 (19)	106 (17)	
• Private practice	295 (29)	48 (12)	247 (40)	
Anaesthesiology subspecialty, *n* (%)				
• No subspecialty	405 (39)	118 (29)	287 (47)	<0.001
• Cardiothoracic and vascular	160 (16)	67 (16)	93 (15)	0.663
• Visceral and urological	248 (24)	146 (35)	102 (17)	<0.001
• Orthopaedic	258 (25)	115 (28)	143 (23)	0.108
• Neurosurgery	143 (14)	47 (11)	96 (16)	0.054
• Gynaecology	196 (19)	100 (24)	96 (16)	0.001
• Paediatric	174 (17)	62 (15)	112 (18)	0.176
• Pain medicine	59 (6)	28 (7)	31 (5)	0.273
• Regional anaesthesia	232 (23)	99 (24)	133 (22)	0.404
• Critical care medicine	99 (10)	72 (17)	27 (4)	<0.001
• Research	37 (4)	22 (5)	15 (2)	0.017

### Opioid-sparing anaesthesia practices

When asked about their level of concern regarding perioperative opioid use, 90% of ESAIC respondents and 83% of ASA respondents indicated they were ‘concerned’ or ‘very concerned’ (*P*<0.001, χ^2^ test of proportions), indicating significantly greater concern among European anaesthesiologists. Although respondents acknowledged the benefits of opioid-sparing anaesthesia (Table 2), its use on a daily basis is lower than 50%. Daily opioid-sparing anaesthesia practice was reported by 37% (95% CI 32–42%) of ESAIC and 40% (95% CI 36–45%) of ASA respondents, with no significant regional difference (*P*=0.064) (Table 2).

**Table 2:** Opioid-sparing anaesthesia practices. ESAIC, European Society of Anaesthesiology and Intensive Care; ASA, American Society of Anaesthesiologists; PONV, postoperative nausea and vomiting.

Variables	Overall (*N*=1028)	ESAIC (*N*=414)	ASA (*N*=614)	*P*-value
Frequency, *n* (%)				0.064
• Never	145 (17)	53 (14)	92 (18)	
• At least once a month	150 (17)	76 (20)	74 (15)	
• At least once a week	244 (28)	108 (29)	136 (27)	
• At least once a day	342 (39)	137 (37)	205 (40)	
Duration, *n* (%)				<0.001
• <6 Months	13 (1.8)	6 (1.9)	7 (1.7)	
• 6 Months to 1 yr	36 (5)	27 (8)	9 (2)	
• 1–3 Yr	178 (24)	103 (32)	75 (18)	
• 3–5 Yr	179 (24)	78 (24)	101 (24)	
• >5 Yr	330 (45)	107 (33)	223 (54)	
Most valuable indications, *n* (%) Appropriate clinical indications				
• Pre-existent opioid-related misuse	345 (34)	141 (34)	204 (33)	0.787
• Chronic opioid user	301 (29)	161 (39)	140 (23)	<0.001
• Chronic pain	283 (28)	164 (40)	119 (19)	<0.001
• High risk of severe postoperative pain	313 (30)	144 (35)	169 (28)	0.016
• Obese patients	364 (35)	137 (33)	227 (37)	0.206
• ASA 3 and 4 patients	145 (14)	56 (14)	89 (15)	0.716
• Older patients	363 (35)	120 (29)	243 (40)	<0.001
• Sleep-related breathing disorders	413 (40)	142 (34)	271 (44)	0.002
• Chronic respiratory insufficiency	309 (30)	126 (30)	183 (30)	0.837
• All patients	236 (23)	97 (23)	139 (23)	0.762
Appropriate surgical indications				
• All surgeries	461 (45)	160 (39)	301 (49)	<0.001
• Bariatric surgery	281 (27)	140 (34)	141 (23)	<0.001
• Oncological surgery	179 (17)	131 (32)	48 (8)	<0.001
• High-risk surgeries	183 (18)	99 (24)	84 (14)	<0.001
Perioperative benefits, *n* (%) Reduction of:				
• PONV	851 (97)	362 (97)	489 (96)	0.107
• Ileus or urinary retention	841 (96)	354 (95)	487 (96)	0.009
• Postoperative morphine requirement	739 (84)	300 (80)	439 (87)	0.040
• Postoperative opioid use disorder	626 (71)	287 (77)	339 (67)	0.004
• Postoperative pain	585 (66)	240 (64)	345 (68)	0.373
Improvement:				
• Postoperative recovery	676 (77)	271 (73)	405 (80)	0.008
• Patient satisfaction	572 (65)	220 (59)	352 (69)	<0.001

Obese patients, older patients, and those with obstructive sleep apnoea were considered the most relevant candidates for opioid-sparing anaesthesia (Table 2). Despite greater concern about the opioid crisis (*P*<0.001), ASA respondents were less likely than ESAIC respondents to promote opioid-sparing anaesthesia for patients with chronic pain or chronic opioid use (*P*<0.001). Interestingly, ASA respondents were more concerned with patient satisfaction outcomes related to opioid-sparing anaesthesia than ESAIC respondents (69% *vs* 59%, *P*<0.004).

**Fig 1:**
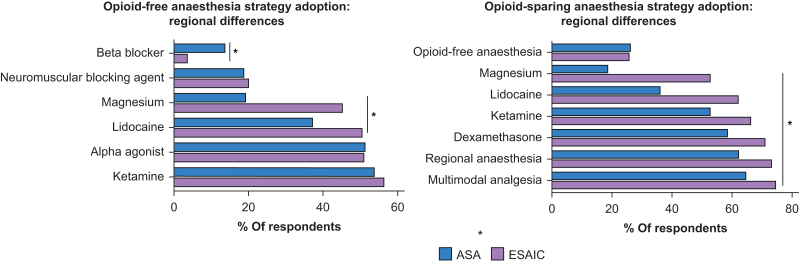
Opioid-sparing anaesthesia and opioid-free anaesthesia strategy adoption: regional differences. ∗*P*<0.001.

Current opioid-sparing anaesthesia practices are detailed in Figure 1. Although ERAS and PSH advocate for the use of opioid-sparing anaesthesia, local protocols were involved in ∼30% of respondent institutions (33% in Europe, 30% in the USA, *P*<0.001).

### Opioid-free anaesthesia practices

**Fig 2:**
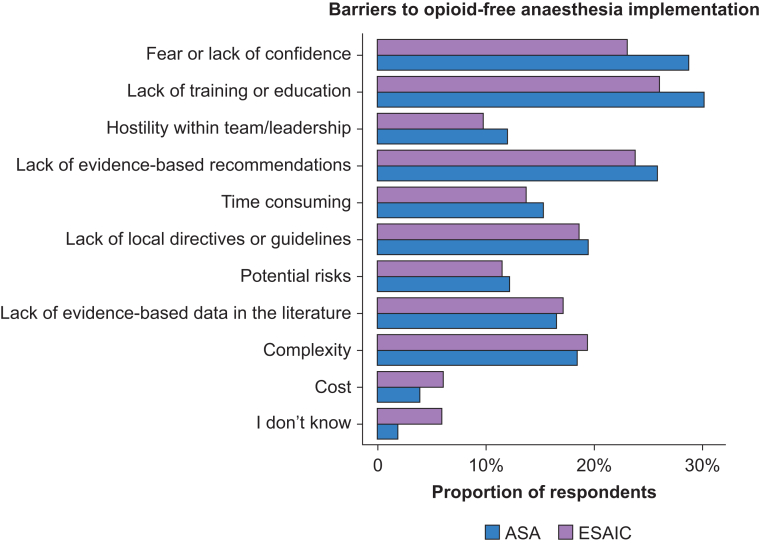
Barriers to opioid-free anaesthesia implementation.

Daily OFA practice was more frequently reported by ASA respondents than by ESAIC respondents (21%, 95% CI 18–25% *vs* 12%, 95% CI 9–15%, *P*<0.001) (Table 3). Approximately 15% of institutions implemented local OFA protocol for specific surgeries. Current OFA practices are detailed in Figure 1. Although 70% of respondents reported at least moderate OFA knowledge, 61% still expressed a need for further training, with no significant regional difference (*P*=0.128). European providers were also more likely to request specific OFA guidelines (81% *vs* 53%, *P*<0.001). Perceived risks associated with OFA are summarised in Table 3. Although ESAIC respondents were more concerned about haemodynamic instability than ASA respondents (43% *vs* 16%, *P*<0.001), patient dissatisfaction and uncontrolled pain were greater concerns among ASA respondents (55% *vs* 30% and 72% *vs* 53%, respectively, *P*<0.001). Barriers to implement OFA practices are presented in Figure 2.

**Table 3:** Opioid-free anaesthesia practices. OFA, opioid-free anaesthesia; PACU, postanaesthesia care unit; ESAIC, European Society of Anaesthesiology and Intensive Care; ASA, American Society of Anesthesiologists.

Variables	Overall (*N*=1028)	ESAIC (*N*=414)	ASA (*N*=614)	*P*-value
OFA frequency, *n* (%)				<0.001
• Never	186 (21)	105 (28)	81 (16)	
• Less than once a month	212 (24)	95 (25)	117 (23)	
• At least once a month	149 (17)	63 (17)	86 (17)	
• At least once a week	182 (21)	67 (18)	115 (23)	
• At least once a day	152 (17)	44 (12)	108 (21)	
Evidence-based benefits published, *n* (%)				<0.001
• I don't know	146 (17)	33 (9)	113 (22)	
• No	110 (13)	60 (16)	50 (10)	
• Neutral	160 (18)	76 (20)	84 (17)	
• Yes	465 (53)	205 (55)	260 (51)	
Evidence-based risks published, *n* (%)				<0.001
• I don't know	193 (22)	58 (16)	135 (27)	
• No	117 (13)	50 (13)	67 (13)	
• Neutral	168 (19)	73 (20)	95 (19)	
• Yes	403 (46)	193 (52)	210 (41)	
Associated risks, *n* (%)				
• Inadequate pain control	663 (65)	220 (53)	443 (72)	<0.001
• Patient dissatisfaction	462 (45)	124 (30)	338 (55)	<0.001
• Haemodynamic instability	272 (27)	177 (43)	95 (16)	<0.001
• Prolonged recovery times in PACU	242 (24)	81 (20)	161 (26)	0.013
• Postoperative patient discomfort	189 (18)	54 (13)	135 (22)	<0.001
• Postoperative delirium	118 (12)	53 (13)	65 (11)	0.277
• Respiratory complications	33 (3)	11 (3)	22 (4)	0.475
• I don't know	65 (6)	33 (8)	32 (5)	0.089

Supplementary Material S5 and S6 detailed factors associated with opioid-sparing anaesthesia and OFA daily use. Regarding opioid-sparing anaesthesia, daily use was significantly more frequent among respondents working in private practice (OR=1.90, *P*=0.002) or perceiving it as beneficial for postoperative recovery (OR=3.48, *P*<0.001). Interestingly, respondents who believed that opioid-sparing anaesthesia improves postoperative recovery were also more likely to report daily use (OR=3.48, *P*<0.001). Younger or less experienced anaesthesia providers were not more likely to use opioid-sparing anaesthesia in daily practice.

Regarding OFA, daily use was significantly more frequent among ASA compared with ESAIC respondents (OR=2.32, *P*=0.002). Working in an academic hospital was associated with lower daily OFA use, compared with private or non-academic hospitals. In contrast to opioid-sparing anaesthesia, greater clinical experience (>10 yr) was significantly associated with daily OFA use (OR=2.65, *P*<0.001). Perceived risks such as inadequate postoperative pain control or patient dissatisfaction were negatively associated with daily OFA adoption.

Exploratory clustering analysis is presented in Supplementary Material S7 revealing three distinct OFA practice profiles: engaged European practitioners (cluster 1), hesitant European Majority (cluster 2), and confident ASA Adopters (cluster 3).

### Content qualitative analysis

Lastly, a total of 180 comments were submitted in the open-ended question (18% of all respondents). Among them, 123 comments referred to OFA practices and 98 to opioid-sparing anaesthesia practices. The distribution of sentiment categories for each anaesthesia strategy is presented in Supplementary Material S8. In addition to sentiment polarity, we performed thematic grouping of responses. The most frequent themes and representative quotes are summarised in Supplementary Material S9. For opioid-sparing anaesthesia, sentiments were predominantly positive (32%) or very positive (5%). In contrast, OFA sentiments were more frequently negative (31%) or very negative (17%). Overall, negative sentiment was more frequently expressed in comments related to OFA practices compared with opioid-sparing anaesthesia (48% *vs* 23%, respectively).

## Discussion

This international survey provides the first comparative insights into the perspectives of anaesthesia providers in Europe and the USA on opioid-sparing anaesthesia and OFA. Despite widespread concerns about perioperative opioid use, daily adoption of opioid alternative strategies remains variable and in many cases, limited. While the survey did not explicitly differentiate the reasons for concern (e.g. adverse effects, risk of persistent use, societal impact), the consistently high level of concern across both regions highlights a shared recognition of the need for safer perioperative opioid stewardship. Only around 40% of respondents reported daily use of opioid-sparing anaesthesia, and fewer than 20% reported using OFA routinely. These rates, along with substantial regional and institutional differences, underscore the complexity of implementing opioid-reducing strategies into daily anaesthetic practice. Importantly, although many respondents expressed concern about perioperative opioid use, this concern did not uniformly translate into routine adoption of opioid-sparing anaesthesia or OFA.

Several potential barriers did emerge, including lack of standardised institutional protocols, limited training, and concerns about patient dissatisfaction or inadequate analgesia. These findings align with prior literature suggesting that opioid-sparing approaches—despite guideline endorsements—may face implementation challenges as a result of logistical, cultural, or educational gaps. We observed notable regional differences. ASA respondents, despite reporting greater concern about the opioid crisis, were not more likely to adopt opioid-sparing strategies than their European counterparts.

This apparent contradiction may reflect a complex interplay of medicolegal concerns, practice culture, and patient expectations—particularly in settings where opioid use is deeply established in perioperative care. Several factors may explain the limited adoption of opioid-sparing practices in ASA members, including both physician and patient-related aspects. First, North America remains the world’s leading region in opioid consumption for pain management.^12^ Importantly, there is no direct evidence that intraoperative opioid avoidance alone can meaningfully reduce the incidence of persistent opioid use or opioid-related harms at the population level. Post-discharge opioid exposure is largely determined by surgical prescribing practices rather than anaesthetic management, as previously reported by our group.^13^ After operation, opioids are still widely prescribed, with 91% of patients receiving them,^14^ even among those managed with opioid-sparing anaesthesia and reporting adequate pain control.^13^ Second, in our survey, 75% of ASA respondents had >10 yr of clinical experience, with potentially limited initial training in opioid-sparing strategies. Although continuing medical education programs have improved in recent years, opioid prescribing habits may persist. Third, greater concern about patient satisfaction among ASA respondents may discourage them from minimising opioid use. Although the Hospital Consumer Assessment of Healthcare Providers and Systems (HCAHPS) pain management items^15^ were removed from reimbursement calculations in 2017, patient satisfaction remains a central concern in USA healthcare delivery. Fourth, USA surgical patients report higher levels of postoperative pain compared with international cohorts,^16^ which may lead providers to favour opioid-based strategies, particularly in complex pain situations.

Daily OFA use was reported less frequently than opioid-sparing anaesthesia and revealed greater regional variability. ASA respondents were more likely than European colleagues to report daily OFA use (21% *vs* 12%), presumably because of higher levels of concern in Europe regarding the haemodynamic instability associated with the use of OFA. Interestingly, providers with >10 yr of clinical experience were significantly more likely to use OFA daily, contrasting with the common narrative that younger practitioners are more likely to adopt innovative approaches. These experienced adopters may be more confident in managing OFA-related challenges or more empowered to implement non-standard protocols. While self-reported knowledge of OFA was moderately high, the majority of respondents indicated a need for additional training. European providers in particular expressed strong demand for formal OFA guidelines (81% of ESAIC respondents *vs* 53% of ASA respondents), highlighting an opportunity for international societies to collaboratively develop consensus recommendations to standardise OFA practices and mitigate safety concerns.

The sentiment analysis of open-ended responses revealed more polarised views toward OFA than toward opioid-sparing anaesthesia. Nearly half of comments on OFA reflected negative sentiment, frequently citing concerns about inadequate pain control and patient dissatisfaction, both of which were also significant barriers to daily use. These perceptions underscore the importance of comprehensive education and outcomes data to guide safe and effective OFA implementation. Patient-centred care and the use of opioid-sparing anaesthesia, however, might be a compromise between safe and efficient use of opioids and opioid sparing,^17^ especially in countries where opioid use after surgery is not problematic.^18^

Our findings also highlight a need for standardised terminology. Despite providing definitions within the survey, respondent interpretations of ‘opioid sparing’ may have varied, particularly in the absence of a clinical vignette or unified protocol. This ambiguity likely affects self-reported prevalence estimates and supports the call for internationally accepted definitions to improve comparability across studies.

Finally, clustering analysis revealed three distinct practice profiles, indicating that adoption is not binary but rather shaped by a spectrum of engagement, institutional support, and regional context.

Our study has several limitations. First, a major limitation was the low overall response rate, particularly among ASA members, which introduces a risk of selection bias, potentially overrepresenting respondents already engaged with opioid-sparing anaesthesia or OFA practices.^19^ In addition, the limited participation from LMIC means that LMIC perspectives are only descriptively reported and cannot be considered representative. We thus included their responses as a separate descriptive subgroup (Supplemental Material S4) to preserve valuable qualitative insight while preventing over- or underestimation of prevalence in the pooled analysis. Together, these limitations mean our results should be interpreted as reflecting the views of a convenience sample of engaged respondents rather than the true global prevalence of opioid-sparing anaesthesia or OFA. While a response rate above 40% is generally recommended,^10^ many recent online anaesthesia surveys have similarly struggled to achieve this.^20^^,^^21^ This may also suggest a possible lower engagement in the topic among ASA members compared with ESAIC members. Consequently, ASA member responses may overrepresent clinicians already convinced by opioid-sparing anaesthesia and OFA. Nevertheless, the high survey completion rate (89%) and rich qualitative data provide valuable insight into current practices and evolving attitudes across two major international anaesthesia communities. Second, the survey was restricted to members of academic societies and had a limited geographical scope. In addition, the survey’s availability only in English and French may have further limited participation from non-English-speaking or non-French-speaking regions, and thus our findings should be interpreted primarily as representing North American and European practices. Furthermore, providers in private practice and early-career respondents were underrepresented. Third, self-reported data may reflect perceived good practices rather than actual daily practices, especially as local OFA and OFA protocol implementation remains rare. Fourth, we did not assess current intraoperative opioid and adjuvant dosages. As many factors may influence these practices, using a standardised clinical case in the survey would have allowed an objective comparison of dose responses among respondents, which can be included in future surveys. Fifth, the qualitative analysis was not conducted by trained qualitative researchers and did not include systematic verbatim coding or thematic saturation procedures.

Despite these limitations, this study offers valuable insight into the current landscape of opioid-sparing anaesthesia practices and provides a foundation for further investigation. Future studies using electronic health record (EHR) data could complement survey-based research by offering objective metrics of opioid use and practice variation.^22^^,^^23^ Moreover, educational initiatives and clinical trials are needed to clarify when, for whom, and how opioid-sparing anaesthesia or OFA strategies should be applied, and what outcomes they meaningfully impact. Lastly, our sample size calculation was not population-based but rather precision-based, which assumes that a sufficient number of respondents would provide an adequately narrow CI. While this approach is commonly used in survey research, it may not fully account for the actual target population size and thus may affect the precision of prevalence estimates.

### Conclusion

Our findings suggest that while interest in opioid-sparing anaesthesia and OFA exists, their daily adoption remains limited and varies widely. Regional perceptions, institutional protocols, and confidence in evidence appear to influence implementation. Standardised terminology and further outcome-driven studies are needed to inform clinical guidelines and foster safe adoption of opioid-reducing strategies.

## Authors’ contributions

Study design/conception: all authors

Study conduct: AJ, YG, TG, YD, BA

Data analysis: TG, YG, AJ, MM

Writing paper: YG, AJ, TG, BA

Editing paper: all authors

Revising paper: all authors

## Declaration of generative AI and AI-assisted technologies in the writing process

The authors used ChatGPT to improve English readability. After using this tool, the authors reviewed and edited the content as needed and take full responsibility for the content of the publication.

## Funding

This study was self-funded. However, NMB is funded by a T32 NHI grant (T32GM148369).

## Declarations of interest

PF is supported by the European Society of Anaesthesiology and Intensive Care (ESAIC) for the Pain and Opioids after Surgery (PANDOS) and the Euro-Periscope Research Groups (IDs ESAIC_GR_2021_PF, ESAIC_RG_PAND, and ESAIC_RG_EP), and received advisory board/speaker fees from Grunenthal, GE Healthcare, and Oncomfort. YG receives speaker fees from GE Healthcare (Buc, France) and received a personal financial support from the Phillip Foundation and Nîmes University Hospital for his UCLA research fellowship. MC and AJ are consultants for Edwards Lifesciences, Irvine, CA, USA. PC receives speaker fees from GE Healthcare (Buc, France). During the past 3 yr, EMPZ received financial support from Gruenenthal for research activities, speakers fees from Gruenenthal and Medtronic, and advisory fees from Merck Sharp & Dohme/Merck and Merz Pharmaceuticals. The other authors declare that they have no conflicts of interest.
