# Supplementary material for: North American and European practices for opioid-sparing and opioid-free anaesthesia: a cross-sectional survey
Source: BJA Open. 2025 Dec 15;16:100511. doi: 10.1016/j.bjao.2025.100511 (PMC12767688; doi:10.1016/j.bjao.2025.100511)
Supplement: Multimedia component 1 [file mmc1.docx]

Online Supplementary Material

- Online Supplementary material S1: Checklist for Reporting Results of Internet E-Surveys (CHERRIES)
- Online Supplementary material S2:
- Online Supplementary material S3: CURRENT TRENDS OF OPIOID SPARING AND OPIOID-FREE ANESTHESIA: AN INTERNATIONAL ONLINE SURVEY
- Online Supplementary material S4: Respondent characteristics – Africa subgroup
- Online Supplementary material S5-S6: Factors associated with daily Opioid sparing anaesthesia practices (S6) and Factors associated with daily OFA practices (S7)
- Online Supplementary material S7-S8: Exploratory Clustering Analysis of OFA User Profiles (S7) and Distribution of comment sentiment for OSA and OFA (S8)
- *Legend S7: Each axis represents a variable, scaled from 0 to 1 (0–100% of the range across clusters). For example, a cluster’s value of 1 on “Training_Need” means it had the highest training need among the three clusters, not necessarily 100% of respondents. “Percent_Europe” reflects the proportion of respondents in each cluster from Europe, also scaled for relative comparison.*
- *Legend S8: Each ring section shows the proportion of comments by sentiment (from very negative to very positive) for each anesthesia strategy. Sentiment coding was based on both manual and automated Qualtrics Text IQ analysis of open-ended responses (Q23). OSA: Opioid-Sparing Anesthesia; OFA: Opioid-Free Anesthesia.*
- Online Supplementary material S9: Most frequent themes and representative quotes
