# Supplementary material for: North American and European practices for opioid-sparing and opioid-free anaesthesia: a cross-sectional survey
Source: BJA Open. 2025 Dec 15;16:100511. doi: 10.1016/j.bjao.2025.100511 (PMC12767688; doi:10.1016/j.bjao.2025.100511)
Supplement: Multimedia component 4 [file mmc4.docx]

**Online Supplemental Material S4: Respondent characteristics – Africa subgroup**

| **Variables** | **Africa**  **(N=73)** |
| --- | --- |
| **Gender**, N (%) |  |
| - Male | 41 (56%) |
| - Female | 32 (44%) |
| **Official tittle**, N (%) |  |
| - CNRA | 4 (6%) |
| - Resident | 3 (4%) |
| - Fellow | 6 (8%) |
| - Attending anaesthesiologist | 30 (41%) |
| - Academic anaesthesiologist | 25 (34%) |
| - Head of Anaesthesia Department | 5 (7%) |
| **Clinical experience**, N (%) |  |
| - In training | 1 (1%) |
| - 0-2 years | 10 (14%) |
| - 3-4 years | 13 (18%) |
| - 5-9 years | 16 (22%) |
| - 10+ years | 33 (45%) |
| **Primary practice environment**, N (%) |  |
| - Academic Teaching Hospital | 45 (62%) |
| - Non-Academic public hospital | 14 (19%) |
| - Private Practice | 14 (19%) |
| **Primary Anesthesiology Subspecialty**, N (%) |  |
| - No Subspecialty | 12 (16%) |
| - Cardiothoracic and Vascular | 15 (21%) |
| - Visceral and Urological | 28 (38%) |
| - Orthopedic | 26 (36%) |
| - Neurosurgery | 10 (14%) |
| - Gynecology | 32 (44%) |
| - Pediatric | 25 (34%) |
| - Pain Medicine | 10 (14%) |
| - Regional Anesthesia | 20 (27%) |
| - Critical Care Medicine | 23 (32%) |
| - Research | 4 (6%) |
| **OSA Frequency**, N (%) |  |
| - Never | 12 (19%) |
| - At least once a month | 17 (26%) |
| - At least once a week | 23 (35%) |
| - At least once a day | 13 (20%) |
| **Duration**, N (%) |  |
| - Less than 6 months | 3 (6%) |
| - 6 months to 1 year | 6 (11%) |
| - 1 to 3 years | 24 (45%) |
| - 3 to 5 years | 10 (19%) |
| - Over 5 years | 10 (19%) |
| **Local Opioid sparing protocol**, N(%) |  |
| - Yes | 15 (23%) |
| **Most valuable indications**, N (%) |  |
| - Pre-existent opioid-related misuse | 12 (16%) |
| - Chronic opioid user | 15 (21%) |
| - Chronic pain | 15 (21%) |
| - High risk of moderate-to-severe postoperative pain | 19 (26%) |
| - Obese patients | 34 (47%) |
| - ASA 3&4 patients | 20 (27%) |
| - Elderly patients | 39 (53%) |
| - Sleep-related breathing disorders | 37 (51%) |
| - Chronic respiratory insufficiency | 39 (53%) |
| - All patients | 10 (14%) |
| - All surgeries | 19 (26%) |
| - Bariatric surgery | 30 (41%) |
| - Oncological surgery | 23 (32%) |
| - High risk surgeries | 18 (25%) |
| **Perioperative benefits**, N (%)  **Reduction:** | |
| - PONV | 63 (97%) |
| - Postoperative ileus or urinary retention | 63 (97%) |
| - Postoperative morphine requirement | 58 (89%) |
| - Postoperative opioid use disorder | 54 (83%) |
| - Postoperative pain | 38 (59%) |
| **Improvement:** |  |
| - Postoperative recovery | 51 (79%) |
| - Patient satisfaction | 37 (57%) |
| **Opioid free anaesthesia (OFA) Frequency,** N (%) |  |
| - Never | 18 (28%) |
| - Less than once a month | 11 (17%) |
| - At least once a month | 9 (14%) |
| - At least once a week | 12 (19%) |
| - At least once a day | 15 (23%) |
| **Evidence-based Benefits Published**, N (%) |  |
| - I don't know | 9 (14%) |
| - No | 5 (8%) |
| - Neutral | 9 (14%) |
| - Yes | 42 (65%) |
| **Evidence-based Risks Published**, N (%) |  |
| - I don't know | 15 (23%) |
| - No | 11 (17%) |
| - Neutral | 11 (17%) |
| - Yes | 30 (43%) |
| **Associated risks,** N (%) |  |
| - Inadequate pain control | 53 (73%) |
| - Patient dissatisfaction | 41 (56%) |
| - Hemodynamic instability | 17 (23%) |
| - Prolonged recovery times in PACU | 17 (23%) |
| - Postoperative patient discomfort | 13 (18%) |
| - Postoperative delirium | 5 (7%) |
| - Respiratory complications | 7 (10%) |
| - I don't know | 3 (4%) |
| **I Need additional OFA training**, N (%) | 56 (86%) |
| **I Need additional OFA guidelines**, N (%) | 60 (92%) |
