# Supplementary material for: North American and European practices for opioid-sparing and opioid-free anaesthesia: a cross-sectional survey
Source: BJA Open. 2025 Dec 15;16:100511. doi: 10.1016/j.bjao.2025.100511 (PMC12767688; doi:10.1016/j.bjao.2025.100511)
Supplement: Multimedia component 5 [file mmc5.docx]

| **Variables** | **Univariable OR (95% CI)** | **p-value** | **Multivariable OR (95% CI)** | **p-value** |
| --- | --- | --- | --- | --- |
| ***Region*** |  |  |  |  |
| - United States vs Europe | 1.17 (0.89, 1.55) | 0.252 | 0.87 (0.61, 1.24) | 0.452 |
| ***Official title*** |  |  |  |  |
| - Academic & Chairmans vs Attending | 1.21 (0.88, 1.66) | 0.245 | 1.54 (1.05, 2.28) | 0.029 |
| - Residents & Fellows vs Attendings | 0.60 (0.38, 0.93) | 0.026 | 0.79 (0.46, 1.36) | 0.397 |
| ***Gender*** |  |  |  |  |
| - Female vs Male | 0.78 (0.58, 1.04) | 0.088 | 0.77 (0.56, 1.05) | 0.095 |
| ***Clinical experience*** |  |  |  |  |
| - Over 10 yrs vs Under 10 yrs | 1.38 (1.03, 1.83) | 0.029 | 0.97 (0.68, 1.38) | 0.861 |
| ***Practice environment*** |  |  |  |  |
| - Non Academic vs Academic hospitals | 1.05 (0.72, 1.53) | 0.794 | 1.10 (0.72, 1.67) | 0.666 |
| - Private practice vs Academic | 1.66 (1.22, 2.27) | 0.001 | 1.90 (1.26, 2.85) | 0.002 |
| **Anesthesia subspeciality** |  |  |  |  |
| - Visceral and Urological: Yes vs No | 1.36 (1.00, 1.84) | 0.049 | 1.31 (0.89, 1.92) | 0.174 |
| - Orthopedic: Yes vs No | 1.35 (1.00, 1.83) | 0.053 | 1.02 (0.70, 1.48) | 0.928 |
| - Pediatric Anesthesia | 0.99 (0.68, 1.41) | 0.933 | 0.87 (0.59, 1.29) | 0.498 |
| ***Perioperative opioid concerns*** |  |  |  |  |
| - Concern vs no concern | 2.30 (1.72, 3.09) | <0.001 | 1.96 (1.43, 2.69) | <0.001 |
| ***PONV reduction concern*** |  |  |  |  |
| - Yes vs No (Reduce PONV) | 4.94 (1.38, 31.5) | 0.035 | 3.55 (0.94, 23.30) | 0.104 |
| **Quality of recovery improvement concern** |  |  |  |  |
| - Yes vs No (Improve Recovery) | 4.32 (2.38, 8.54) | <0.001 | 3.48 (1.85, 7.05) | <0.001 |

**Online Supplementary Material S5: Factors associated with daily Opioid sparing anaesthesia practices**

OR: odds ratio; CI: confidence interval

**Online Supplementary Material S6: Factors associated with daily OFA practices**

OFA: opioid free anaesthesia; OR: odds ratio; CI: confidence interval

| **Variables** | **Univariable OR (95% CI)** | **p-value** | **Multivariable OR (95% CI)** | **p-value** |
| --- | --- | --- | --- | --- |
| ***Region*** |  |  |  |  |
| - United States vs Europe | 2.03 (1.40, 2.99) | <0.001 | 2.32 (1.38, 3.97) | 0.002 |
| ***Official title*** |  |  |  |  |
| - Academic & Chairmans vs Attending | 1.04 (0.69, 1.54) | 0.853 | 1.19 (0.73, 1.94) | 0.485 |
| - Residents & Fellows vs Attendings | 0.16 (0.05, 0.38) | <0.001 | 0.42 (0.12, 1.20) | 0.136 |
| ***Gender*** |  |  |  |  |
| - Female vs Male | 0.78 (0.53, 1.14) | 0.206 | 0.97 (0.63, 1.48) | 0.901 |
| ***Clinical experience*** |  |  |  |  |
| - Over 10 yrs vs Under 10yrs | 3.86 (2.46, 6.30) | <0.001 | 2.65 (1.58, 4.64) | <0.001 |
| ***Practice environment*** |  |  |  |  |
| - Non Academic vs Academic hospitals | 1.88 (1.16, 3.01) | 0.01 | 1.79 (1.03, 3.08) | 0.036 |
| - Private practice vs Academic hospitals | 2.48 (1.67, 3.70) | <0.001 | 1.69 (1.02, 2.80) | 0.042 |
| **Anesthesia subspeciality** |  |  |  |  |
| - Visceral and Urological: Yes vs No | 1.41 (0.96, 2.05) | 0.079 | 1.01 (0.61, 1.67) | 0.973 |
| - Orthopedic: Yes vs No | 1.98 (1.37, 2.86) | <0.001 | 1.86 (1.15, 2.97) | 0.011 |
| - Pediatric Anaesthesia | 1.63 (1.05, 2.48) | 0.025 | 1.39 (0.85, 2.23) | 0.185 |
| **Associated risk of inadequate pain control** |  |  |  |  |
| - Yes vs No (Inadequate Pain) | 0.53 (0.36, 0.77) | 0.001 | 0.38 (0.23, 0.61) | <0.001 |
| **Associated risk of Hemodynamic instability** |  |  |  |  |
| - Yes vs No (Haemodynamic Instability) | 0.77 (0.51, 1.12) | 0.182 | 0.94 (0.59, 1.48) | 0.796 |
| **Associated risk of patient dissatisfaction** |  |  |  |  |
| - Yes vs No (Patient Dissatisfaction) | 0.67 (0.47, 0.95) | 0.024 | 0.62 (0.40, 0.96) | 0.033 |
| **Not practiced OFA:**  **Lack of training/education** |  |  |  |  |
| - Yes vs No (Lack of Training) | 1.57 (1.10, 2.24) | 0.012 | 1.84 (1.19, 2.85) | 0.006 |
| **Not practiced OFA:**  **Fear or lack of confidence** |  |  |  |  |
| - Yes vs No (Lack of Confidence) | 1.28 (0.89, 1.84) | 0.180 | 1.16 (0.74, 1.81) | 0.518 |
