# Supplementary figures and images for: North American and European practices for opioid-sparing and opioid-free anaesthesia: a cross-sectional survey
Source: BJA Open. 2025 Dec 15;16:100511. doi: 10.1016/j.bjao.2025.100511 (PMC12767688; doi:10.1016/j.bjao.2025.100511)

## Slide 1
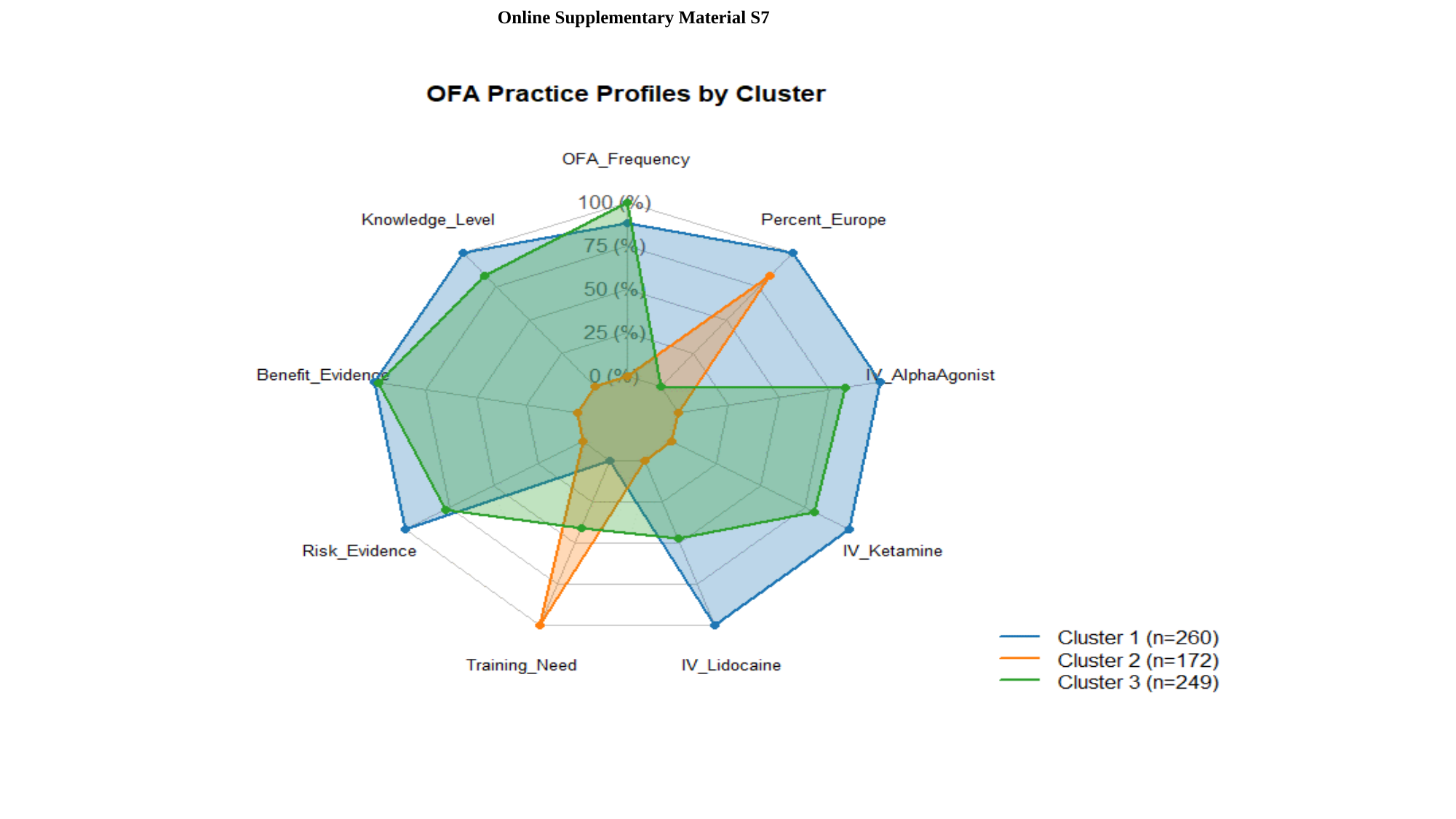

Online Supplementary Material S7

## Slide 2
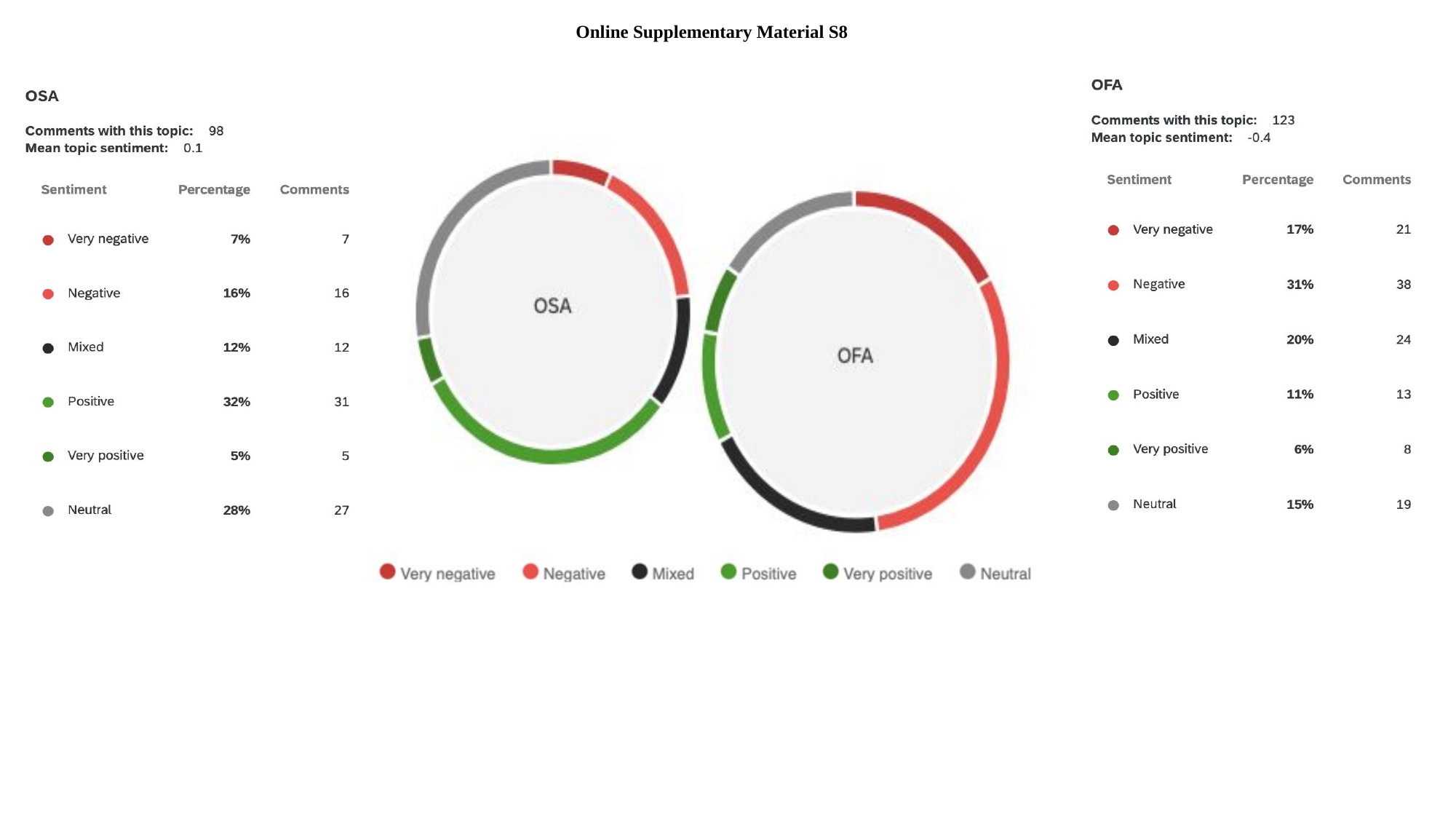

Online Supplementary Material S8

Supplement: Multimedia component 6 [file mmc6.pptx]
