# Supplementary material for: North American and European practices for opioid-sparing and opioid-free anaesthesia: a cross-sectional survey
Source: BJA Open. 2025 Dec 15;16:100511. doi: 10.1016/j.bjao.2025.100511 (PMC12767688; doi:10.1016/j.bjao.2025.100511)
Supplement: Multimedia component 7 [file mmc7.pdf]

SUPPLEMENTAL MATERIAL 3: CURRENT TRENDS OF OPIOID-SPARING AND OPIOID-FREE ANESTHESIA: AN INTERNATIONAL ONLINE SURVEY

Dear Members,

Dear Members,
We invite you to participate in a survey aimed **at better understanding current anesthetic practices in opioid-sparing anesthesia and opioid-free anesthesia (OFA) in surgery.**
This survey consists of 26 questions and is estimated to take **between 5 and 10 minutes to complete**. All anesthesiology professionals, including residents, fellows, attending anesthesiologists, and Certified Registered Nurse Anesthetists are encouraged to participate.

Please fill out the questionnaire only once, even if you receive multiple reminders. By clicking on the link, you agree to participate. You may stop or exit the survey at any time, but you will not be able to return to the initial form.

Participating in this survey does not expose you to any risks: participation is **voluntary and anonymous**. Refusal or choice not to participate will not result in any penalty. The information collected in this study will not be used or distributed for future projects. All data will be stored on the RedCap™ online software, which is securely protected by a password.
If you have any questions or comments regarding this study, you may contact the principal investigator, Dr. Yann Gricourt, MD, at yann.gricourt@chu-nimes.fr, Department of Anesthesia at Nîmes University Hospital, France, or the co-investigator, Dr. Alexandre Joosten, MD, PhD, at AJoosten@mednet.ucla.edu.

Have you already completed this questionnaire?

- Yes, partially, but I did not finish it
- Yes, I have already completed it fully
- No, I have never completed it

START THE SURVEY

Section 1: General Information a)

Q1: What is your official title?

- CRNA (nurse anesthetist)
- Resident (medical doctor in training)
- Anesthesiology fellow
- Attending anesthesiologist (board-certified specialist)
- Academic anesthesiologist (assistant professor, full professor)
- Head of Anesthesia Department (i.e Chair)

Q2: How would you describe your gender?

- Female
- Male
- Other
- Decline to state

Q3: How many years have you been in practice (post-training)?

- 0-2 years
- 3-4 years
- 5-9 years
- 10+ years
- Still in training (i.e residency)

Q4: What is your primary practice environment?

- Academic / Teaching Hospital
- Non-Academic public hospital
- Private Practice

Q5: In which country are you currently practicing?

- List of countries

Section 2: Opioid-Sparing Anesthesia (OSA) Practices

Q6: How concerned are you about the use of opioids during the perioperative period in your clinical practice?

- Not at all concerned
- Slightly concerned
- Neutral
- Somewhat concerned
- Very concerned
- I don’t know

If “Not at all concerned”: skip to Q23 and complete the survey.
If other items: continue the survey.

Q7: Please indicate your primary concerns:

- Opioid-related side effects (e.g., constipation, sedation, postoperative nausea and vomiting: Yes, No, I don’t know.
- Awareness of the opioid epidemic and its societal impact: Yes, No, I don’t know.
- Concerns about opioid tolerance and dependence: Yes, No, I don’t know.
- Consideration of patient-specific factors (e.g., age, comorbidities) : Yes, No, I don’t know.
- Interest in alternative pain management approaches: Yes, No, I don’t know.
- Adherence to institutional or regulatory guidelines: Yes, No, I don’t know.
- Personal clinical experience: Yes, No, I don’t know.
- Availability of non-opioid analgesic options: Yes, No, I don’t know.

Opioid-Sparing Anesthesia (OSA) is an approach to managing perioperative and postoperative nociception/pain with the goal of minimizing opioid use. The term "opioid-sparing" implies reducing exposure to opioid medication such as Remifentanil, Sufentanil, Fentanyl, Hydromorphone, or Morphine.

Q8: Does your institution or group have a written protocol, care guide, or local directive concerning OSA in this setting?

- Yes
- No
- I don’t know

Q9: In the past month, how often have you offered an OSA strategy in your clinical practice?

- Never
- At least once a month
- At least once a week
- At least once a day

If “Never”, please skip directly to the questions on patient outcomes (Q11 and Q12).
If “at least once a month”, “at least once a week” or “at least once a day”: continue the survey.

Q9-1: If you have integrated OSA strategies into your practice, for approximately how long have you been doing so?

- Less than 6 months
- 6 months to 1 year
- 1 to 3 years
- 3 to 5 years
- Over 5 years

Q10: What OSA strategies do you routinely use? Please select all that apply.

- Complementary Regional Anesthesia (neuraxial anesthesia, peripheral nerve block, parietal block, etc.)
- IV magnesium
- IV ketamine
- IV lidocaine
- IV dexamethasone
- Multimodal postoperative analgesia (paracetamol, NSAID) approach
- An opioid-free anesthesia approach
- None of the above

Q11-a: In your opinion, in which context would an OSA strategy be the most valuable? Please select up to 5 patient characteristics where you believe an OSA strategy would be most clinically relevant.

- Pre-existent opioid-related misuse
- Chronic opioid user
- Chronic pain
- High risk of moderate-to-severe postoperative pain
- Obese patients
- ASA 3&4 patients
- Elderly patients
- Sleep-related breathing disorders
- Chronic respiratory insufficiency
- All patients' whatever comorbidities or surgeries
- None of these patients
- I don't know

Q11-b: In your opinion, in which context would an OSA strategy be the most valuable? Please select up to 3 types of surgeries where you believe an OSA strategy would be most clinically relevant.

- High risk surgeries (cardiothoracic, vascular, and major abdominal surgeries)
- Oncological surgery
- Bariatric surgery
- Orthopedic surgery (excluding exclusive regional anesthesia)
- All types of surgeries
- None of these surgeries
- I don't know

Q12: What perioperative benefits would be associated with the use of an OSA strategy?

- Reduction of postoperative nausea and vomiting: Yes, No, I don’t know.
- Reduction of postoperative pain: Yes, No, I don’t know.
- Reduction of postoperative morphine requirement: Yes, No, I don’t know.
- Reduction of postoperative ileus and/or urinary retention: Yes, No, I don’t know.
- Reduced incidence of postoperative opioid use disorder: Yes, No, I don’t know.
- Improvement in postoperative recovery: Yes, No, I don’t know.
- Improvement in postoperative patient satisfaction: Yes, No, I don’t know.

Section 3: Opioid-Free Anesthesia (OFA) Practices

Q13: How would you define Opioid-Free Anesthesia (OFA)? Please, select all that apply

- No intraoperative opioids
- Reduction of intraoperative opioids
- Balanced anesthesia combining multiple drugs and adjuvants
- Inability to titrate morphine in the recovery room
- No postoperative opioid prescription
- I do not know

Q14: What is your current knowledge level on OFA?

- Very Low
- Low
- Moderate
- High

Q15: In the past year, how often have you practiced OFA in your clinical practice?

- Never
- Less than once a month
- At least once a month
- At least once a week
- At least once a day

If "Never" to Q15, proceed to Q15-a:
Q15-a: What are the reasons you have not practiced OFA in your clinical practice over the past year? Select all that apply:

- Cost
- Potential risks
- Complexity
- Time consuming
- Fear or lack of confidence
- Lack of training or education
- Lack of evidence-based recommendations
- Lack of evidence-based data in the literature
- Lack of local directives or locally used guidelines in this context
- Hostility within my team or from my department leadership
- I don't know

Q16: To your knowledge, has evidence-based proof of OFA benefits been published?

- I don’t know
- No, not at all
- No, probably not
- Neutral
- Yes, probably
- Yes, very likely

Q17: To your knowledge, has evidence-based proof of OFA risks been published?

- I don’t know
- No, not at all
- No, probably not
- Neutral
- Yes, probably
- Yes, very likely

Q17.1: Please specify the types of risks you associate with the use of OFA. Please, select all that apply

- Respiratory complications
- Inadequate pain control
- Hemodynamic instability (bradycardia, arterial hypotension, etc.)
- Postoperative patient discomfort (dizziness, PONV, shivering, etc.)
- Postoperative delirium or cognitive impairment
- Prolonged recovery times (in PACU, hospital length of stay, etc.)
- Patient dissatisfaction
- I don’t know

If “Less than once a month,” “At least once a month,” “At least once a week,” or “At least once a day” to Q15, proceed to Q18 and Q19:
Q18: Which drugs for OFA do you use most frequently in your daily clinical practice? Excluding multimodal analgesics and regional anesthesia if applicable. Please select all that apply.

- IV Lidocaine
- IV Ketamine
- IV Magnesium
- IV Alpha agonist drug (clonidine or dexmedetomidine)
- IV Beta blocker drug
- IV Neuromuscular blocking agent

Q19: Do you have an OFA written protocol in your institution?

- Yes
- No
- I do not know

Q20: What are the main barriers or obstacles that you perceive to the use of OFA? Please, select all that apply

- Cost
- Potential risks
- Complexity
- Time consuming
- Fear or lack of confidence
- Lack of training or education
- Lack of evidence-based recommendations
- Lack of evidence-based data in the literature
- Lack of local directives or locally used guidelines in this context
- Hostility within my team or from my department leadership
- I don’t know

Section 4: Education and Acceptance

Q21: Would you need, for your practice, additional training or education in OFA?

- Yes
- No
- I don’t know

Q22: In your opinion, is there a need for international guidelines to outline appropriate OFA use during the perioperative period?

- Yes
- No
- I don’t know

Section 1 : General Information b)

Q23: If any, what subspecialty of anesthesiology do you primarily practice? Please, select all that apply

- No subspecialty in particular
- Cardiothoracic and Vascular Anesthesia
- Anesthesia for Visceral and Urological Surgery
- Anesthesia for Orthopedic Surgery
- Anesthesia for Neurosurgery
- Anesthesia for Gynecology
- Pain Medicine
- Pediatric Anesthesia
- Regional Anesthesia
- Critical Care Medicine
- Research

Do you have any comments or feedback related to the topics discussed (OFA, opioid-sparing strategies, etc.)?

FINISH
