# Supplementary material for: North American and European practices for opioid-sparing and opioid-free anaesthesia: a cross-sectional survey
Source: BJA Open. 2025 Dec 15;16:100511. doi: 10.1016/j.bjao.2025.100511 (PMC12767688; doi:10.1016/j.bjao.2025.100511)
Supplement: Multimedia component 8 [file mmc8.docx]

| **Theme** | **Frequency (% of comments)** | **Representative Quote** |
| --- | --- | --- |
| Need for opioid free anaesthesia (OFA) training/guidelines | 28% | “OFA should be taught systematically during residency; I don’t feel confident using it daily.” |
| Concern about inadequate pain control | 24% | “I am hesitant to avoid opioids entirely because of risk of uncontrolled pain in PACU.” |
| Concern about haemodynamic instability | 15% | “I stopped using OFA because patients often required high vasopressor support.” |
| Positive experience with opioid sparing anaesthesia | 14% | “Multimodal opioid-sparing techniques have improved recovery profiles in my patients.” |
| Logistical barriers (drug availability, institutional support) | 12% | “Dexmedetomidine is not available in my hospital, limiting OFA implementation.” |
| Patient satisfaction and acceptance | 7% | “Patients expect opioids and complain if they feel pain despite OFA.” |

**Online Supplementary Material S9: Most frequent themes and representative quotes**

OFA: opioid free anesthesia; PACU: post-anesthesia care unit
